# Dietary Administration Effects of Exopolysaccharide Produced by *Bacillus tequilensis* PS21 Using Riceberry Broken Rice, and Soybean Meal on Growth Performance, Immunity, and Resistance to *Streptococcus agalactiae* of Nile tilapia (*Oreochromis niloticus*)

**DOI:** 10.3390/ani13203262

**Published:** 2023-10-19

**Authors:** Nantaporn Sutthi, Eakapol Wangkahart, Paiboon Panase, Thipphiya Karirat, Sirirat Deeseenthum, Nyuk Ling Ma, Vijitra Luang-In

**Affiliations:** 1 Department of Agricultural Technology, Faculty of Technology, Mahasarakham University, Maha Sarakham 44150, Thailand; nantaporn.s@msu.ac.th (N.S.); eakapol.w@msu.ac.th (E.W.); 2 Applied Animal and Aquatic Sciences Research Unit, Division of Fisheries, Faculty of Technology, Mahasarakham University, Maha Sarakham 44150, Thailand; 3 Unit of Excellence Physiology and Sustainable Production of Terrestrial and Aquatic Animals (FF66-UoE014), School of Agriculture and Natural Resources, University of Phayao, Phayao 56000, Thailand; tong33_panamagigas@hotmail.com; 4 Fisheries Division, School of Agriculture and Natural Resources, University of Phayao, Phayao 56000, Thailand; 5 Natural Antioxidant Innovation Research Unit, Department of Biotechnology, Faculty of Technology, Mahasarakham University, Maha Sarakham 44150, Thailand; thipphiya.k@gmail.com (T.K.); sirirat.d@msu.ac.th (S.D.); 6 Faculty of Science and Marine Environment, Universiti Malaysia Terengganu, Kuala Nerus 21030, Terengganu, Malaysia; nyukling@umt.edu.my

**Keywords:** antimicrobial activity, antioxidant, *Bacillus tequilensis*, exopolysaccharide, functional feed ingredient, immunomodulation, *Oreochromis niloticus*

## Abstract

**Simple Summary:**

Overuse of antibiotics has resulted in antibiotic-resistant bacteria and adverse changes in aquaculture ecology. Thus, natural bioproducts have been sourced for use in aquaculture as substitutes for antibiotics. This study investigated bacterial exopolysaccharide (EPS) as an antioxidant and a stimulant for fish immune systems. Agro-industrial biowaste carbon sources that are more economical than sugars are preferred to mitigate the production cost of EPS for commercial applications. In this study, the EPS produced from riceberry broken rice and soybean meal by *Bacillus tequilensis* PS21 bacteria from milk kefir grain within 72 h displayed antioxidant activity and antimicrobial activity toward pathogenic bacteria *Streptococcus agalactiae*. In Nile tilapia (*Oreochromis niloticus*), EPS supplementation at a high dose of 2.0 g EPS/kg feed significantly increased fish survival post-challenge with *S. agalactiae* EW1 and also boosted the fish’s immune system. The study suggested a method for utilizing agro-industrial biowaste as a value-added EPS source for a bio-circular green economy model to preserve a healthy environment while also enabling sustainable aquaculture.

**Abstract:**

Overuse of antibiotics in aquaculture has generated bacterial resistance and altered the ecology. Aquacultural disease control requires an environmentally sustainable approach. Bacterial exopolysaccharides (EPSs) as bioimmunostimulants have not been extensively explored in aquaculture. This study investigated EPS produced from 5% *w*/*v* riceberry broken rice as a carbon source and 1% *w*/*v* soybean meal as a nitrogen source by *Bacillus tequilensis* PS21 from milk kefir grain for its immunomodulatory, antioxidant activities and resistance to pathogenic *Streptococcus agalactiae* in Nile tilapia (*Oreochromis niloticus*). The FTIR spectrum of EPS confirmed the characteristic bonds of polysaccharides, while the HPLC chromatogram of EPS displayed only the glucose monomer subunit, indicating its homopolysaccharide feature. This EPS (20 mg/mL) exhibited DPPH scavenging activity of 65.50 ± 0.31%, an FRAP value of 2.07 ± 0.04 mg FeSO_4_/g DW, and antimicrobial activity (14.17 ± 0.76 mm inhibition zone diameter) against *S. agalactiae* EW1 using the agar disc diffusion method. Five groups of Nile tilapia were fed diets (T1 (Control) = 0.0, T2 = 0.1, T3 = 0.2, T4 = 1.0, and T5 = 2.0 g EPS/kg diet) for 90 days. Results showed that EPS did not affect growth performances or body composition, but EPS (T4 + T5) significantly stimulated neutrophil levels and serum lysozyme activity. EPS (T5) significantly induced myeloperoxidase activity, catalase activity, and liver superoxide dismutase activity. EPS (T5) also significantly increased the survival of fish at 80.00 ± 5.77% at 14 days post-challenge with *S. agalactiae* EW1 compared to the control (T1) at 53.33 ± 10.00%. This study presents an efficient method for utilizing agro-industrial biowaste as a prospective source of value-added EPS via a microbial factory to produce a bio-circular green economy model that preserves a healthy environment while also promoting sustainable aquaculture.

## 1. Introduction

The aquaculture industry in Thailand has expanded over the last two decades, accompanied by tangible socioeconomic growth, with the country rated among the top twenty-five globally in 2023 for fisheries produce [1]. *Oreochromis niloticus*, also known as Nile tilapia, is now a valuable aquaculture species as a result of rapid expansion, with excellent survival and reproductive rates in captivity, producing highly-priced premium meat grade [1]. Over 4,827,581 tons of this fish were farmed in over 120 countries in 2021 [1]. However, Nile tilapia farming is impacted by *Streptococcus agalactiae* infections, resulting in significant global financial losses [2]. Inhibiting and treating such diseases is possible due to the widespread application of antibiotics [3]. However, this has led to the proliferation of antibiotic-resistant bacteria, with residual antimicrobial compounds in fisheries products, environmental risks, and shifts in aquaculture ecosystems [4]. Thus, copious research has been undertaken to develop an environmentally friendly technique for aquacultural disease management, focusing on identifying alternative natural bioproducts as substitutes for antibiotics.

In recent decades, feed additive supplementation has gained popularity as a reasonably priced option in the aquafeed business [5] that also improves fish development, their immunological system, and nutrition efficacy [6]. Polysaccharides, both intracellular and extracellular, are among the many beneficial substances that certain bacteria produce and expel. Monosaccharides and non-carbohydrate compounds like proteins, phosphate, as well as nucleic acids combine to form exopolysaccharides (EPSs), which are high-molecular-weight polymers [7]. Microbial EPSs are immunostimulants, very highly biodegradable, and safer than chemicals. However, the influence of EPS structure and origin on biological activities such as immunomodulation in fish is poorly understood [8]. Previously, we showed that *Bacillus tequilensis* PS21 of Thai milk kefir [9] produced EPS with antioxidant and anti-tyrosinase activities using lactose as a carbon source [10]. Alternative lower-cost carbon sources are preferred to reduce the production cost of EPS for commercial purposes. In Thailand, with ever-increasing market demand for agricultural products, most agricultural biowastes (e.g., sugarcane molass, cassava molass, starch molass, broken rice, coffee grounds, coconut water, copra meals, and fruit wastes) at several million tons per year often end up in municipal waste streams and are not efficiently utilized. In Thailand, one of the most widely available varieties of rice is the riceberry cultivar, which is prized for its high antioxidant content. Twenty to thirty percent of riceberry rice product output (1200 to 1800 tons every harvest season) is lost as broken rice due to the polishing procedure. Thai farmers produce vast quantities of riceberry broken rice (RBR), which are sold as cheap raw materials for animal feeds or as ingredients in purees, snacks, and Chinese noodles [11].

The use of EPS from agro-biowastes in aquaculture for fish feed is underexplored and requires further investigation. To date, how bacterial EPSs modulate the immune system is not well understood, with very few reports on the efficacy of bacterial EPS as a fish health-promoting feed additive [7,12,13,14]. No previous research has examined the efficacy of microbial EPS as a feed additive for Nile tilapia. Thus, this study investigated whether *B. tequilensis* PS21 could convert RBR agro-industrial biowastes into bioactive EPS products as a functional feed element for Nile tilapia growth, the stimulation of immune-related gene expression, innate immunity, and disease tolerance against pathogenic bacteria to promote sustainable aquaculture.

## 2. Materials and Methods

### 2.1. Bacterial Cultivation

As previously mentioned [9,10], *B. tequilensis* PS21 (GenBank: MN844072.1) was derived from Thai milk kefir. The bacteria were grown in Tryptic Soy Broth (Oxoid, Basingstoke, UK) at pH 7.0 for 24 h while being shaken at 37 °C and 150 rpm. After adjusting the culture suspension to an OD_600nm_ of 0.1, it was inoculated into fresh RBR media for EPS generation during a 72 h period [15].

### 2.2. Bacterial EPS Production

RBR as a carbon source was derived from the Agrarian Network of E-San Enterprise, Roi Et Province, Thailand. As a nitrogen source, soybean meal (SBM) was bought from the Friends of Agriculture shop in Kantarawichai District, Maha Sarakham Province, Thailand. RBR and SBM were powdered to a fine 200 mm mesh and kept dry. Following that, RBR (5% *w*/*v*) and SBM (1% *w*/*v*) were combined in 100 mL of distilled water (adjusted to pH 7.0) and sterilized at 110 °C, 15 psi for 15 min. Microbial seed inoculum (3% *v*/*v*) was introduced into RBR plus SBM media and cultivated for 72 h in triplicate at 37 °C and 150 rpm agitation speed [15].

### 2.3. Crude EPS Extraction and Compositional Analysis

Crude EPS was isolated from *B. tequilensis* PS21 cells using ice-cold ethanol precipitation as previously described [12,16] with some modifications. Extraction of EPS was performed by spinning the culture at 10,000 g for 20 min to remove the bacterial cell pellet. The supernatant was combined with 200 mL of cold ethanol overnight at 4 °C. EPS was precipitated by centrifuging at 10,000× *g* for 20 min, then rinsing twice with distilled water, drying at 40 °C until reaching a constant weight, and then powdering before storing in a desiccator. Lowry’s method [17] was used to quantify the protein content of EPS (20 mg EPS/mL in Milli-Q water) using bovine serum albumin as a standard. EPS carbohydrate content was assessed using the phenol-sulfuric acid method with D-glucose as the standard [18], and EPS nucleic acid content was measured at A_260nm_, as previously reported [19]. Experimental diets with crude EPS powder were used as a feed component. Bioactive assays were conducted using a 20 mg/mL stock solution of EPS powder dissolved in deionized water.

### 2.4. Monosaccharide Composition Analysis

A sealed tube containing 5 mL of 2 M trifluoroacetic acid (TFA) was used for hydrolyzing 100 mg of EPS at 100 °C for 6 h. After neutralizing with 1 N NaOH, the hydrolysate was filtered using a 0.22 μm syringe. An HPLC (LC-20 AD, RID-10 A refractive index detector, Shimadzu, Japan) with an Aminex HPX-87H column (Bio-Rad, Hercules, CA, USA) at 65 °C was used to assess EPS monomers [10]. The mobile phase was H_2_SO_4_ (0.005 M) at 0.5 mL/min for 40 min per sample with 10 μL injection volume. EPS sugars were identified using a UV detector with reference to monosaccharide sugar standards (Sigma-Aldrich, St. Louis, MO, USA).

### 2.5. Fourier-Transform Infrared Spectroscopy (FTIR) Analysis

The chemical bond characteristics of EPS was recorded using a Fourier transform infrared (FTIR) microscope Spectrum GX (PerkinElmer Inc., Waltham, MA, USA). Desiccated EPS (1 mg) was ground in KBr particles (20 mg) in a 1:20 *w*/*w* ratio, and the resulting sample was analyzed using FTIR between 400 and 4000 cm^−1^.

### 2.6. Scanning Electron Microscopic (SEM) Analysis

The morphological characteristics of EPS were observed using an SEM (Leo/1450 Carl Zeiss, Oberkochen, Germany) at 15 kV. The samples were sized appropriately and deposited on a conductive film after being properly desiccated. Morphological characteristics of samples were observed at various magnifications.

### 2.7. Antioxidant Activity Assays

#### 2.7.1. Scavenging Ability on 2,2-Diphenyl-1-picrylhydrazyl (DPPH•) Free Radicals

A protocol was conducted as previously reported [20,21]. Decolorization of a methanol solution of 2,2-diphenyl-1-picrylhydrazyl (DPPH) was used to determine the hydrogen atom-donating capacity of EPS. In methanol solution, DPPH produces a violet/purple hue that diminishes to yellow in the presence of antioxidants. DPPH solution (10 mM) in methanol (180 μL) was combined with 20 μL of a 20 mg/mL EPS extract. The reaction mixture was vigorously vortexed and left for 30 min in the dark at room temperature. At 517 nm, the absorbance of the mixture was measured spectrophotometrically. Dextran served as the standard for EPS. The following equation was used to derive the percentage of DPPH radical scavenging activity:

% DPPH radical scavenging activity = {(A_0_ − A_1_)/A_0_} × 100%

where A_0_ is the absorbance of the control, and A_1_ is the absorbance of the EPS extract.

#### 2.7.2. Ferric Ion Antioxidant Reducing Power (FRAP)

A protocol was conducted as previously reported [22]. The FRAP reagent was made by combining acetate buffer (300 mM, pH 3.6), 2,4,6-tri[2-pyridyl]-s-triazine (10 mM in 40 mM HCl), and FeCl_3_·6H_2_O (20 mM) in a 10:1:1 ratio, respectively. The FRAP reagent (180 μL) was combined with EPS (20 μL of a 20 mg/mL stock solution) and vigorously agitated. After 30 min, the absorbance was read at 593 nm. Calibration was performed using an aqueous FeSO_4_ solution with known concentrations. Dextran served as the standard for EPS. The FRAP values (mg FeSO_4_/g DW) were calculated.

#### 2.7.3. Scavenging Ability on Hydroxyl Radicals

A protocol was conducted as previously reported [23]. Principally, the Fe^3+^-ascorbate-EDTA-H_2_O_2_ system (Fenton reaction) generated the hydroxyl radical. The assay relies on the quantification of the 2-deoxy-D-ribose degradation product, which produces a pink chromogen upon heating with thiobarbituric acid (TBA) at low pH. The reaction mixture contained 0.8 mL of phosphate buffer solution (50 mM, pH 7.4), 0.2 mL of EPS extract (20 mg/mL), 0.2 mL of EDTA (1.04 mM), 0.2 mL of FeCl_3_ (1 mM), and 0.2 mL of 2-deoxy-D-ribose (28 mM). The mixture was maintained in a 37 °C water immersion, and the reaction was initiated by adding 0.2 mL of ascorbic acid (2 mM) and 0.2 mL of hydrogen peroxide, H_2_O_2_ (10 mM). After 1 h incubation at 37 °C, 1.5 mL of chilled TBA (10 g/L) and 1.5 mL of 25% HCl were added, and the reaction mixture was heated at 100 °C for 15 min before chilling with water. The absorbance of the solution was measured at 532 nm using a spectrophotometer. The hydroxyl radical scavenging capacity of the antioxidant was determined by measuring the inhibition of 2-deoxy-D-ribose oxidation by hydroxyl radicals. The percentage of hydroxyl radical scavenging activity was determined using the following formula:

% hydroxyl radical scavenging activity = [A_0_ − (A_1_ − A_2_)] × 100/A_0_

where A_0_ is the absorbance of the control without a sample, A_1_ is the absorbance after mixing the sample with 2-deoxy-D-ribose, and A_2_ is the absorbance of the sample without 2-deoxy-D-ribose.

### 2.8. Antimicrobial Activity of EPS

The pathogenic bacterium used in this experiment was *S. agalactiae* EW1 (GenBank: OR272051.1), isolated from infected Nile tilapia in Northeast Thailand. Using microbiologically standard protocols, the bacterium was identified as *S. agalactiae*, consisting of catalase-negative Gram-positive cocci with β-hemolytic properties. The biochemical analyses were conducted in accordance with the manufacturer’s instructions using the API 20 STREP system (BioMerieux, Craponne, France). The bacterium was confirmed by PCR analysis using universal primers for the 16S rRNA gene as previously described [24].

An agar disc diffusion assay was conducted by placing discs (4 mm) containing 100 μL of EPS (20 mg/mL) on LB agar plates inoculated with *S. agalactiae* EW1 (10^6^ CFU/mL). After 24 h incubation at 37 °C, the diameter of the clear inhibition zone (mm) surrounding each disc was recorded in triplicate. Dextran (20 mg/mL) was used as an EPS standard to compare the effect of EPS.

### 2.9. Trial Fish Diet Formula

Five different doses of EPS powder were used to create the trial fish diet (T1 (Control) = 0.0, T2 = 0.1, T3 = 0.2, T4 = 1.0, and T5 = 2.0 g EPS/kg). The basal diet consisting of fish meal, soybean meal, corn, broken rice, rice bran, as well as vitamins and minerals was a commercially available feed from Betagro^®^, Thailand, containing 32% crude protein, 12% ash, 6% fiber, and 4% crude fat. The combined meal was coated with 4% agar after using 20 g of guar gum as a pellet binder. Proximate analyses of diets were carried out [25].

### 2.10. Experimental Design

The treatments followed a completely randomized design (CRD) in this trial. Four hundred and fifty juvenile male Nile tilapia were derived from Maha Sarakham Inland Fisheries Research and Development Center (Thailand) and acclimatized for two weeks in 12 fiberglass tanks (1500 L/tank) with feeding twice daily. Then, the fish (14.87 ± 0.56 g weight and 7.63 ± 0.06 cm length) were randomly divided into five groups (*n* = 30 fish/group/replicate): T1 = basal diet without EPS; T2 = EPS 0.1 g/kg diet; T3 = EPS 0.2 g/kg diet; T4 = EPS 1.0 g/kg diet; and T5 = EPS 2.0 g/kg diet). The fish received trial diets at 5% body weight twice a day for 90 days. Water at 26.54 ± 1.17 °C, dissolved oxygen at 4.81 ± 1.48 mg/L, total ammonia nitrogen at 0.45 ± 0.65 mg/L, and pH value at 7.69 ± 0.54 were maintained to ensure water quality with 80% water replaced weekly.

### 2.11. Sample Collections

After the trial ended, the fish were anesthetized with clove oil (100 mg/L), and 0.5 mL of caudal vein blood was collected (6 samples/treatment) for hematological analysis in anticoagulant tubes for serum separation from blood. Spleen and liver specimens of thirty fish (6 samples/treatment) were dissected, washed in phosphate-buffered saline buffer (PBS), kept in RNA*later*^®^ (Thermo Fisher Scientific, Waltham, MA, USA) in the fridge overnight, and stored at −20 °C.

### 2.12. Growth Measurements

After the trial ended, all fish were determined for growth rate using the mathematical growth model as follows: Weight gain (WG; g) = final weight (g) − initial weight (g); Length gain (LG; cm) = final length (cm) − initial length (cm); Average daily gain (ADG; g/day) = [final weight (g) − initial weight (g)]/days; Specific growth rate (SGR; %/day) = 100 × [{ln final weight (g) − ln initial weight (g)}/days]; Feed conversion ratio (FCR) = total feed (g)/weight gain (g); Survival rate (SR, %) = [number of survived fish/initial number of fish] × 100.

### 2.13. Body Chemical Composition and Organosomatic Indices

For approximate analysis, fish fillets (*n* = 3/treatment) were determined using the AOAC (1995) technique [25]. After drying in an oven at 40 °C for 72 h, the moisture content was estimated, with crude protein at N × 6.25 using the Kjeldahl technique [25]. Ash proportion was assessed using a muffle furnace for 4 h at 600 °C, while crude lipid percentage was examined using the Soxhlet method [25]. Organosomatic indices such as %fillet, %carcass, hepatosomatic index (%HSI), viscerosomatic index (%VSI), and spleenosomatic index (SSI; %) were computed as follows: Fillet (%) = [100 × (fillet weight (g)/body weight)]; Carcass (%) = [100 × (carcass weight (g)/body weight)]; Hepatosomatic index (%HSI) = [100 × (liver weight (g)/body weight)]; Viscerosomatic index (%VSI) = [100 × (visceral weight (g)/body weight)]; Spleenosomatic index (SSI; %) = [100 × (spleen weight (g)/body weight)].

### 2.14. Blood Chemical Analysis and Antioxidant Enzyme Activity

The blood specimens were transported to the Vet Central Lab in Khon Kaen, Thailand, for blood chemical analysis. The concentration of malondialdehyde (MDA) in serum was evaluated by detecting thiobarbituric acid reactive substances (TBARS) using a previous method [26]. Liver superoxide dismutase (SOD) activity was assessed using a previous method [27]. Briefly, 20 μL of liver homogenate was mixed with 940 μL sodium carbonate buffer (pH 10.2, 0.05 M) and 40 μL epinephrine (30 mM dissolved by adding 30 μL of HCl, Sigma-Aldrich, St. Louis, MO, USA). The inhibition of epinephrine auto-oxidation in the alkaline medium to adrenochrome was recorded after 30 and 90 s at 480 nm using a GENESYS™ 20 visible spectrophotometer (Thermo Fisher Scientific, Dreieich, Germany). A control was prepared as 960 μL sodium carbonate buffer and 40 μL epinephrine. SOD activity was calculated using the following formulas:

The percent of inhibition (%) = 100 − ((ΔA control − ΔA sample/ΔA control) × 100). SOD activity (U/g liver) = % inhibition × 3.75.

Catalase activity (CAT) in the liver was calculated [28] with the differences in absorbance recorded after 20 s (A_1_) and after 80 s (A_2_) of incubation at 240 nm at room temperature. The CAT value was calculated as (A_1_ − A_2_)/0.0008.

### 2.15. Hematological Parameters

A hematological examination was performed on the collected blood samples at the Vet Central Lab in Khon Kaen, Thailand. Hematocrit (Hct), hemoglobin, and other parameters were calculated following previous methods [29,30]. A Neubauer hemocytometer was used to count the erythrocytes (RBC) and leukocytes (WBC). Blood was dispersed and stained with a combination of Giemsa and May–Grunwald for differential leucocyte count measurement. Hematocrit (Hct) was determined using heparinized micro-hematocrit capillary tubes after centrifugation (12,000 rpm for 5 min) and reported as percentages. Hemoglobin (Hb) concentration was determined using a lysing reagent solution (ABX reagent, Horiba, Montpellier, France) for erythrocyte lysis and cyanide-free hemoglobin determination. All of the heme irons were oxidized and stabilized, and the hemoglobin was liberated. Using a hematology analyzer (ABX Micros EVS 60, Horiba, Montpellier, France), the resultant complexes were quantified by spectrophotometry with a wavelength of 550 nm. Mean cell volume (MCV) = Hct(%) × 10/RBCs (10^6^ mm^−3^); Mean cell hemoglobin (MCH) = Hb (g/dL) × 10/RBCs count (10^6^ mm^−3^); Mean corpuscular hemoglobin concentration (MCHC) = Hb (g/dL)/Hct (%).

### 2.16. Immunological Analysis

Serum lysozyme activity was determined by lysing the lysozyme-sensitive Gram-positive bacterium *Micrococcus lysodeikticus* (Sigma-Aldrich, St. Louis, MO, USA) and observing the resulting turbidity [31]. The myeloperoxidase (MPO) activity present in serum was measured following the previous method [27]. Briefly, 20 µL of serum was diluted with phosphate-buffered saline (PBS) in 96-well plates. Next, 35 µL of 20 mM 3,3′,5,5′-tetramethylbenzidine hydrochloride (Sigma-Aldrich, St. Louis, MO, USA) was added along with 35 µL of 5 mM hydrogen peroxide (H_2_O_2_) (Merck, Darmstadt, Germany). After 2 min, 35 µL of 4 M sulfuric acid (H_2_SO_4_) was added to stop the reaction. A_450nm_ was recorded using an iMark^TM^ absorbance microplate reader (Bio-Rad, Hercules, CA, USA).

### 2.17. Serum Bactericidal Activity

Serum bactericidal activity was performed following a previous method [32]. Briefly, at 90 days after feeding the experimental diet, the serum of fish (*n* = 3 /treatment) was taken. One hundred microliters of *S. agalactiae* EW1 (10^3^ CFU/mL) from 10-fold serial dilution in 0.9% sterile saline was mixed with 100 µL of fish serum in each group and then incubated at 30 °C for 1 h. The numbers of *S. agalactiae* EW1 were estimated by viable counts with the agar plate-spread method on tryptic soy agar (TSA) 24 h after incubation.

### 2.18. Gene Expression

The spleen and liver total RNA were extracted according to the guidelines provided by the PureLink RNA Mini Kit (Thermo Fisher Scientific, Waltham, MA, USA). Gene expression analysis was performed by the Chiang Mai, Thailand-based company Animals Molecular Diagnostic Services (AMDS) Limited. Quantification of two transcripts (interleukin-1 beta (*IL-1β*) and tumor necrosis factor-alpha (*TNF-α*)) was performed in the spleen and liver with beta-actin (*β-actin*) serving as an endogenous reference (Table 1). The amplification was operated via PCRmax Eco 48 Real-Time qPCR System (PCRmax, Staffordshire, UK) at the following protocol: 95 °C for 10 min; 40 cycles of denaturation for 15 s at 95 °C; annealing for 15 s at the melting temperature (T_m_) of 58 °C; and extension at 72 °C for 10 s. The 2^−∆∆CT^ method approach [33] was then used to analyze the data for relative expression.

### 2.19. Challenge Test

Ten healthy fish from each replicate (*n* = 30/treatment) were moved to an *S. agalactiae* EW1 challenge study after the feeding trial concluded. The previous protocol [33] was used to prepare the bacterial suspension. Intraperitoneal injections of a bacterial dosage of 10^8^ CFU/mL in sterile saline solution (0.9%) were given to the challenged fish. The preliminary work showed that using a bacterial dosage of 10^8^ CFU /mL for the bacterial challenge was appropriate. This was previously reported [34].

The percentage of surviving tilapia in each treatment was determined 14 days post-challenge. Survival rate (%) = [number of survived fish/initial number of fish] × 100.

The relative percentage of survival (RPS) was calculated [35]. RPS (%) = 100 × [1 − (% mortality of EPS treated group/% mortality of control group)].

### 2.20. Statistical Analysis

Duncan’s post hoc test was used in all data to compare treatments after one-way analysis of variance (ANOVA). Except for the challenge test, Dunnett’s multiple comparisons test was used for statistical analysis. Significance was set at *p* < 0.05, with mean ± SD values represented.

## 3. Results

### 3.1. EPS Characterization

**Figure 1:**
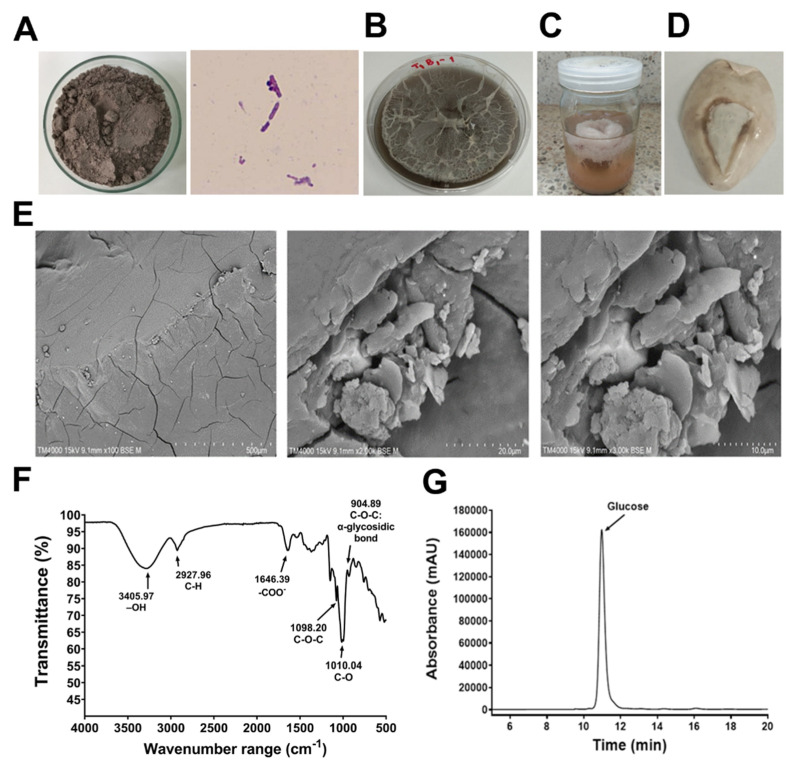
Characteristics of EPS. (**A**) RBR powder as carbon source substrate for EPS production by *B. tequilensis* PS21, (**B**) EPS production on RBR-containing agar, (**C**) EPS production in RBR-containing liquid medium, (**D**) Crude EPS, (**E**) SEM morphological analysis of EPS (**F**) FTIR spectroscopy analysis of EPS and (**G**) HPLC sugar compositional analysis of EPS hydrolysate (only peak of glucose at 10.98 min).

Microscopic observation showed that the colonies of *B. tequilensis* PS21 were Gram-positive rods (Figure 1A) and were able to utilize RBR powder (Figure 1A) for EPS production, as previously reported [13]. EPS appeared whitish and spongy on agar containing 5% *w*/*v* RBR (Figure 1B) and appeared whitish, fluffy, and spongy floating on the surface of the liquid culture containing 5% RBR plus 1% SBM (Figure 1C). After ethanol precipitation and air drying, the unrefined EPS appeared lustrous and creamy whitish in color (Figure 1D) and exhibited a compact structure and smooth surfaces without pores under 100× magnification of SEM and a flake-like structure with an uneven grainy surface under 2000× and 3000× magnifications (Figure 1E). The FTIR spectra of EPS showed a polyhydroxilic compound with a large absorption band at 3405 cm^−1^ on the stretching vibration of hydroxyl groups of links (OH) (Figure 1F). Stretching C-H bonds created the bands at 2927 and 1646 cm^−1^. The principal absorption bands that described the α (1 → 6) EPS were detected in the range of 1098 cm^−1^ and connected with the vibrations of the glycoside link C–O–C, while 904 cm^−1^ denoted the alpha (α) conformation of this link. The absorption band occurrence at 1010 cm^−1^ demonstrated high flexibility of the polysaccharide chain (Figure 1F). HPLC sugar compositional analysis of EPS hydrolysate displayed only a peak of glucose at 10.98 min, indicating its homopolysaccharide feature (Figure 1G).

Regarding the compositional analysis, the highest carbohydrate content of 404.23 ± 9.86 mg/g EPS was recorded (76.58%) and was significantly different (*p* < 0.05) from nucleic acid content (107.92 ± 4.58 mg/g EPS, 20.45%) and protein content (15.66 ± 0.61 mg/g EPS, 2.97%) (Table 2). EPS production by *B. tequilensis* PS21 using 5% RBR and 1% SBM was 2.40 ± 0.20 g EPS/100 mL at 72 h.

### 3.2. EPS Bioactivities

The DPPH radical was used as a test to investigate the antioxidative properties of antioxidants. The DPPH scavenging activity of *B. tequilensis* PS21 (65.50 ± 0.31%) was slightly lower than that of dextran (73.98 ± 0.27%) at the same concentration of 20 mg/mL. The FRAP assay was used to evaluate the reducing antioxidant activity. Here, FRAP values of both EPS (2.07 ± 0.04 mg FeSO_4_/g DW) and dextran (2.23 ± 0.04 mg FeSO_4_/g DW) were similar (Table 3). Likewise, the hydroxyl radical is a highly reactive oxidant that reacts with almost any molecule [36]. Both EPS and dextran exhibited similar scavenging activities against hydroxyl radicals (80.53 ± 0.87% and 80.94 ± 0.46%, respectively) (Table 3). These results suggested that dextran had slightly better antioxidant activities based on three assays than EPS from *B. tequilensis* PS21.

**Figure 2:**
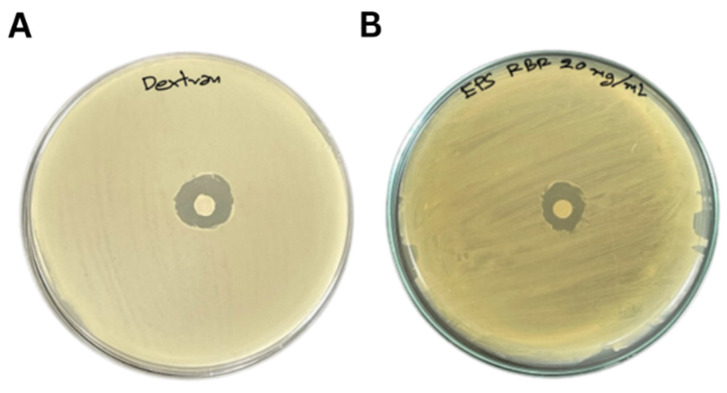
Antimicrobial activity against *S. agalactiae* EW1 using an agar disc diffusion. (**A**) Dextran (20 mg/mL) and (**B**) EPS (20 mg/mL).

Antibacterial activity of EPS and dextran against *S. agalactiae* EW1 is shown in Table 2 and Figure 2. The inhibition zone of EPS (14.17 ± 0.76 mm) was lower than that of dextran (15.50 ± 0.05 mm).

### 3.3. Growth Performances

Proximate analysis of the trial meals (T1–T5) showed that their chemical compositions were similar in amounts of crude protein, crude fat, moisture, and ash (Table 4). Nile tilapia growth responses to EPS supplementation were indifferent (*p* > 0.05) (Table 5).

### 3.4. Hematological and Blood Chemical Profiles

When compared to the T1-control diet, the neutrophil count increased considerably (*p* < 0.05) when EPS was supplemented at 1.0 and 2.0 g/kg (T4 and T5). No statistically significant differences were found between treatments in terms of red blood cell count, white blood cell count, hematocrit, hemoglobin, lymphocytes, eosinophils, monocytes, MCH, MCV, or MCHC. Total protein, globulin, albumin, alanine aminotransferase, aspartate aminotransferase, cholesterol, and blood urea nitrogen all showed no significant treatment-by-treatment differences (*p* > 0.05) (Table 6).

### 3.5. Body Composition and Organosomatic Indices

The fillet values, carcass weight, and percentage of crude lipid, crude protein, ash, and moisture were all similar across treatments (*p* > 0.05) (Table 7). Similar results were found when looking at organosomatic indices (HIS, VSI, and SSI) (*p* > 0.05) (Table 7).

### 3.6. Blood Biochemical Profiles and Immunological/Antioxidant Parameters

Blood chemical profiles showed no differences among treatments (Table 8). However, after 90 days of feeding, fish exposed to EPS at doses of 1.0–2.0 g/kg (T4 and T5) displayed highly enhanced serum lysozyme activity more than the control group (T1) (Table 9).

Similarly, T4 and T5 groups had significantly greater CAT activity in comparison with the control group (Table 9). MPO activity was statistically greater (*p* < 0.05) in T5-fed fish compared to control fish (Table 9). The T5 group had the greatest value of liver SOD activity; however, no statistical variations in MDA levels amongst treatments (*p* > 0.05) were observed (Table 9).

### 3.7. Gene Expressions in the Spleen and Liver

**Figure 3:**
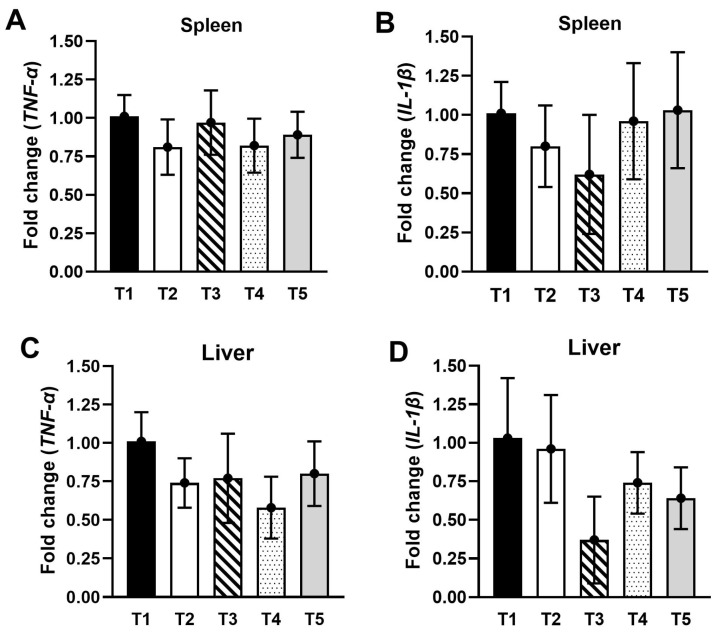
Effect of EPS for 90 days on immune gene expressions in the spleen and liver of Nile tilapia (*O. niloticus*). (**A**) spleen *TNF-α*, (**B**) spleen *IL-1β*, (**C**) liver *TNF-α*, (**D**) liver *IL-1β*. Data are given as mean ± SD.

The effects of EPS supplement on the gene expression of cytokines in Nile tilapia spleen and liver showed no significant differences in interleukin-1 beta (*IL-1β*) and tumor necrosis factor-alpha (*TNF-α*) gene expressions in fish fed with EPS diets (Figure 3A–D).

### 3.8. Bactericidal Activity

**Figure 4:**
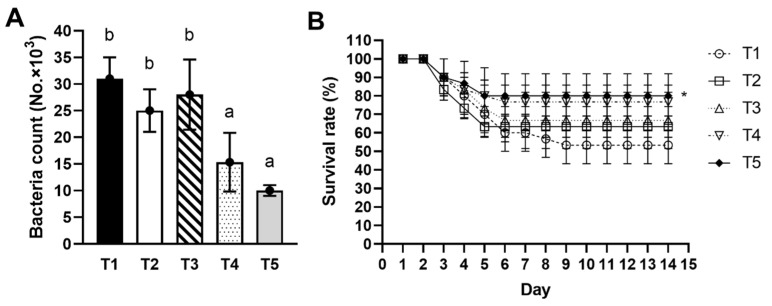
Effect of EPS on *S. agalactiae* EW1 infection. (**A**) *S. agalactiae* EW1 counts after incubation with serum of Nile tilapia (*O. niloticus*) treated with different EPS doses, (**B**) Percentage of survival rate of Nile tilapia treated with different EPS doses for 14 days post-challenge test with *S. agalactiae* EW1. Data are given as mean ± SD, and different superscripts indicate significant differences (*p* < 0.05). An asterisk (*) shows statistical significance compared to the control (T1) based on Dunnett’s multiple comparisons test analysis.

Significantly lower bacterial *S. agalactiae* EW1 counts (*p* < 0.05) after incubation with fish serum were found in fish fed 1.0 and 2.0 g/kg diet (T4 and T5) compared to T3, T2 and T1-control diets (Figure 4A).

### 3.9. Pathogen Challenge Test

The resistance of Nile tilapia to *S. agalactiae* EW1 infection was examined as a function of dietary EPS supplementation. On day 3, some fish appeared dead and displayed signs of fin erosion, blood loss, and bulging eyes. Survival rates for the T1 to T5 groups were 53.33 ± 10.00%, 63.33 ± 5.77%, 66.66 ± 15.27%, 76.66 ± 15.27%, and 80.00 ± 5.77%, respectively (Figure 4B). The mean protective efficacy in terms of relative percent of survival (RPS) compared to the control (T1) was T5 (57.14%), followed by T4 (50.00%), T3 (28.57%), and T2 (21.43%). Fish supplied with 2.0 g/kg of EPS (T5) were the most resistant to *S. agalactiae* EW1, and only the T5 group gave a significantly higher survival rate (*p* < 0.05) in comparison with the control (T1) (Figure 4B).

## 4. Discussion

The emergence of pathogens and the widespread use of antibiotics for disease treatment have presented numerous challenges and environmental concerns to aquaculture over the past decade [37]. Natural bioactive compounds have been sought after for use as feed additives to promote fish health because of their lower toxicity and eco-friendliness [38]. Probiotic strains from different habitats are gaining attention owing to their distinctive characteristics and structures that they impart to EPSs, with antibacterial, antibiofilm, anti-inflammatory, and antioxidant properties [39]. Nevertheless, how bacterial EPSs modulate the immune system remains poorly understood, with little known about the proficiency of EPSs for use as a feed additive to promote fish health [7,12,13,14], and no research on the effectiveness of microbial EPSs as a feed additive for Nile tilapia.

This study investigated Nile tilapia growth, innate immune system, immune gene expressions, and disease tolerance following dietary administration of EPS produced by *B. tequilensis* PS21 using 5% RBR and 1% SBM as carbon and nitrogen sources, respectively. Using agro-biowastes as substrates, *B. tequilensis* PS21 produced a high EPS content of 2.40 g EPS/100 mL after 72 h. This is the first report showing the use of RBR combined with SBM to produce EPS by *B. tequilensis* PS21 and applying it as a feed additive in aquaculture.

EPS production by *B. tequilensis* was significantly higher than 0.788 g EPS/L production by *B. velezensis* KY498625 using molasses and yeast extract as carbon and nitrogen sources, respectively [40]. Differences in EPS production result from different bacterial strains, genetic makeups, substrates, medium ingredients, and bacterial growth conditions. In this study, *Bacillus* EPS showed similar antioxidant activities to dextran based on FRAP assay and hydroxyl scavenging activity assay, probably due to the same glucose monomer subunits comprising the homopolysaccharide. However, the DPPH scavenging activity assay confirmed that dextran had stronger antioxidant activity, which may contribute to stronger antimicrobial activity against *S. agalactiae* EW1. This difference between *Bacillus* EPS and dextran results from their diverse detailed polysaccharide structures. Molecular weight, monosaccharide concentration, and glycosidic bond arrangement are all important in determining the antioxidant activity of polysaccharides [41].

This work showed for the first time that *Bacillus* EPS exerted antimicrobial activity toward a bacterial pathogen of Nile tilapia, *S. agalactiae* EW1. The antibacterial processes of microbial EPSs have been linked to interfering with the cell division machinery of bacteria by influencing the composition of the membrane or respiratory chain of a bacterial cell. To exert their antibacterial effect, microbial EPSs likely combine with oligopeptides in Gram-positive bacteria or acyl-homoserine lactone in Gram-negative bacteria [42,43,44]. Effects of *Bacillus* EPS (0.1–2.0 g/kg diet) on Nile tilapia growth performances were examined. Results showed that different EPS doses did not affect growth performances or body composition. However, using EPS of 2.0–3.0 g/kg feed isolated from *Ganoderma lucidum* mycelium as a functional feed element on red hybrid Tilapia (*Oreochromis* sp.) resulted in notable growth enhancement [45]. Aligned with this, Hu et al. (2023) [39] showed that EPS of 0.1 and 0.5 g/kg feed from *Lactiplantibacillus plantarum* HMX2 greatly enhanced juvenile Turbot, *Scophthalmus maximus*, growth, while Mozambique tilapia (*O. mossambicus*) ingesting feed supplemented with EPS from *B. licheniformis* and EPS-mediated zinc oxide nanoparticles (EPS-ZnO NPs) at 2.0–10.0 g/kg feed showed improved growth performance [12]. *Bacillus* EPS was unable to enhance Nile tilapia growth probably because of the absence of certain EPS characteristics needed to stimulate such growth, the low doses of EPS used in this study, or different types of fish used in previous reports compared to our study. Serum biochemical and innate immunological markers could be used to assess the effect of feed additives on fish physiology and health [46]. Liver function and nutrition metabolism are often evaluated through ALT, ALP, total protein, glucose, and albumin [47]. In this study, *Bacillus* EPS had no impact on liver function and nutrient metabolism. EPS of 1.0–2.0 g/kg feed (T4 and T5) significantly stimulated neutrophil level and serum lysozyme activity, while EPS of 2.0 g/kg feed (T5) significantly induced MPO, CAT and SOD activity levels.

Cellular-immunological characteristics are the main defense of fish against harmful microorganisms [48]. To protect cells from harmful compounds, antioxidant enzymes like glutathione peroxidase (GPx), SOD, and CAT scavenge superoxide anions and convert hydrogen peroxide into water and oxygen, respectively [49]. Both SOD and CAT levels increased with EPS supplementation at 2.0 g/kg diet, demonstrating its protective impact. These results correlated with serum MPO activity increases in *Oncorhynchus mykiss* fed *B. subtilis* [50] and catla fingerlings fed *B. amyloliquefaciens* [51]. These findings concurred with previous results. In Mozambique tilapia (*O. mossambicus*) on day 30, groups fed with EPS and EPS-ZnO NPs (10 g/kg) showed enhanced lysozyme, SOD, and CAT activity levels (*p <* 0.05) [12]. 

An *in vitro* antimicrobial attribute of EPS toward *S. agalactiae* EW1 using the agar disc diffusion method was also in accordance with an *in vivo* challenge test of Nile tilapia against *S. agalactiae* EW1 in animal experiments. EPS (2.0 g/kg diet) greatly enhanced fish survival rate at 80% for a 14-day post-challenge test with *S. agalactiae* EW1. Microbial EPSs may disrupt bacterial cell division [42,43,44], corroborating the effects of EPSs on pathogen challenge tests in recent studies. *Aeromonas hydrophila* and *Vibrio parahaemolyticus* outbreaks in Mozambique tilapia farming were alleviated by *Bacillus* EPSs and EPS-ZnO NPs [12]. Similarly, *Cyprinus carpio* L. were protected from ailments caused by *A. hydrophila* when administered with EPS from *Lactococcus lactis* Z-2 [13].

## 5. Conclusions

This is the first report documenting the use of *B. tequilensis* PS21 EPS from RBR and SBM agro-biowastes as a natural functional aquafeed component to enhance the antioxidant content and immunity of Nile tilapia (*O. niloticus*). EPS demonstrated anti-*S. agalactiae* activity *in vitro* and in a pathogen-infected *in vivo* setting. These findings support EPS application as an immunostimulant for fish wellness enhancement. Polysaccharides are unlikely to have an impact on bacterial resistance to front-line antibiotics and are also regarded as being affordable, safe, and biodegradable [52]. EPS produced by bacteria exhibited numerous advantages over EPS from animals and plants, including robust operability, high reproductive efficiency, and superior performance [53]. This study presents a practical approach to converting agro-industrial biowaste into EPS with added value using a microbial factory as part of a bio-circular green economy model to protect the environment while also promoting sustainable aquaculture.

## Figures and Tables

**Table 1:** PCR primer sequences used for quantitative real-time gene expression analysis.

Gene	Accession No.	Primer Sequence (5′→3′)	Tm (°C)	Product Size (bp)
*β* *-actin*	EF206801	Forward: GCTACTCCTTCACCACCACAG Reverse: CGTCAGGCAGCTCGTAACTC	58	138
*IL-1β*	JF957370	Forward: TGCACTGTCACTGACAGCCAA Reverse: ATGTTCAGGTGCACTTTGCGG	58	144
*TNF-α*	AY428948	Forward: CCAGAAGCACTAAAGGCGAAGA Reverse: CCTTGGCTTTGCTGCTGATC	58	82

**Table 2:** Compositions of EPS and its production at 72 h.

Sample	Content (mg/g EPS)			EPS Production (g/100 mL)
	Carbohydrate	Protein	Nucleic Acid	
EPS	404.23 ± 9.86 ^a^ (76.58%)	15.66 ± 0.61 ^c^ (2.97%)	107.92 ± 4.58 ^b^ (20.45%)	2.40 ± 0.20

Distinct lowercase superscripts in the row show significant differences (*p* < 0.05).

**Table 3:** DPPH, FRAP, hydroxyl antioxidant activities of EPS and its antimicrobial activity against *S. agalactiae* EW1.

Sample	DPPH Radical Scavenging Activity (%)	FRAP Value (mg FeSO_4_/g DW)	Hydroxyl Radical Scavenging Activity (%)	Inhibition Zone (mm Diameter)
EPS (20 mg/mL)	65.50 ± 0.31 ^b^	2.07 ± 0.04 ^a^	80.53 ± 0.87 ^a^	14.17 ± 0.76 ^b^
Dextran (20 mg/mL)	73.98 ± 0.27 ^a^	2.23 ± 0.04 ^a^	80.94 ± 0.46 ^a^	15.50 ± 0.05 ^a^

Distinct lowercase superscripts in the columns show significant differences (*p* < 0.05).

**Table 4:** Proximate analysis of the five diets (% dry matter) with EPS supplementation.

Chemical Composition	T1 (0.0 g/kg)	T2 (0.1 g/kg)	T3 (0.2 g/kg)	T4 (1.0 g /kg)	T5 (2.0 g/kg)
Crude protein (%)	32.68	32.16	32.38	31.97	32.75
Crude lipid (%)	4.13	4.17	4.29	4.74	4.31
Crude fiber (%)	4.47	4.35	4.29	4.61	4.48
Moisture (%)	12.23	12.95	12.82	13.08	12.73
Ash (%)	10.09	9.80	9.87	10.17	9.98
NFE (%) ^1^	48.63	49.53	49.18	48.51	48.47
GE (MJ/kg) ^2^	17.71	17.75	17.79	17.76	17.77

^1^ Nitrogen-free extract (NFE%) = 100 − (crude protein + crude lipid + ash + crude fiber). ^2^ Gross energy (GE) was calculated based on 23.6, 39.5, and 17.2 kJ/g protein, lipid, and carbohydrates, respectively.

**Table 5:** Growth performances and survival rate of Nile tilapia (*O. niloticus*) fed diets supplemented with EPS for 90 days.

Parameter	T1 (0.0 g/kg)	T2 (0.1 g/kg)	T3 (0.2 g/kg)	T4 (1.0 g/kg)	T5 (2.0 g/kg)
Initial body weight (IBW; g)	15.23 ± 0.73	14.90 ± 0.34	15.20 ± 0.95	15.16 ± 1.19	15.20 ± 1.01
Initial length (IL; cm)	7.63 ± 0.15	7.56 ± 0.23	7.66 ± 0.11	7.73 ± 0.30	7.60 ± 0.17
Final body weight (FBW; g)	57.61 ± 2.73	57.81 ± 3.29	60.01 ± 3.03	61.75 ± 1.37	60.22 ± 2.20
Final length (FL; cm)	11.40 ± 0.09	11.41 ± 0.26	11.60 ± 0.43	11.73 ± 0.12	11.76 ± 0.20
Weight gain (WG; g)	42.39 ± 2.19	42.90 ± 3.08	44.83 ± 2.30	46.60 ± 1.67	45.00 ± 1.33
Length gain (LG; cm)	3.77 ± 0.15	3.83 ± 0.05	3.90 ± 0.34	4.00 ± 0.36	4.20 ± 0.10
Average daily growth gain (ADG; g/day)	0.68 ± 0.03	0.69 ± 0.05	0.72 ± 0.04	0.75 ± 0.03	0.73 ± 0.02
Specific growth rate; SGR (%/day)	2.14 ± 0.05	2.18 ± 0.07	2.22 ± 0.05	2.26 ± 0.13	2.21 ± 0.06
Feed conversion ratio; (FCR)	1.46 ± 0.03	1.51 ± 0.06	1.53 ± 0.04	1.47 ± 0.11	1.52 ± 0.20
Survival rate (%)	83.33 ± 6.66	88.88 ± 6.94	85.55 ± 6.93	92.22 ± 6.94	88.89 ± 10.71

Data are given as mean ± SD.

**Table 6:** Hematological values of Nile tilapia (*O. niloticus*) fed diets supplemented with EPS for 90 days.

Parameter	T1 (0.0 g/kg)	T2 (0.1 g/kg)	T3 (0.2 g/kg)	T4 (1.0 g/kg)	T5 (2.0 g/kg)
RBC (×10^6^ cells/mm^3^)	2.10 ± 0.06	1.96 ± 0.11	2.10 ± 0.16	2.11 ± 0.29	2.02 ± 0.13
Hemoglobin (g/dL)	11.00 ± 2.98	9.53 ± 3.30	10.50 ± 1.73	11.66 ± 1.62	10.73 ± 0.66
Haematocrit (%)	33.00 ± 9.64	27.66 ± 9.50	31.00 ± 6.24	34.66 ± 4.16	31.33 ± 1.52
WBC (×10^3^ cells/mm^3^)	5.82 ± 1.46	4.14 ± 0.82	5.91 ± 1.80	6.39 ± 3.31	6.55 ± 1.10
Neutrophils (%)	30.00 ± 5.29 ^b^	34.00 ± 1.58 ^b^	32.66 ± 7.50 ^ab^	38.66 ± 1.52 ^a^	39.00 ± 1.73 ^a^
Eosinophils (%)	1.33 ± 0.57	1.16 ± 0.28	1.00 ± 0.01	1.16 ± 0.76	1.33 ± 0.57
Lymphocytes (%)	62.00 ± 4.58	63.66 ± 7.09	65.00 ± 5.19	66.66 ± 5.85	68.66 ± 2.51
Monocytes (%)	2.66 ± 0.57	3.66 ± 1.52	4.66 ± 1.52	3.00 ± 1.00	4.00 ± 0.01
MCV (fL)	175.00 ± 1.00	169.00 ± 4.35	171.33 ± 1.52	168.66 ± 6.80	174.66 ± 2.51
MCH (pg)	54.96 ± 0.80	57.33 ± 5.00	57.76 ± 2.08	57.00 ± 1.01	57.50 ± 2.52
MCHC (g/dL)	31.43 ± 0.65	33.86 ± 2.11	33.70 ± 1.15	33.86 ± 0.90	32.86 ± 1.35

Data are given as mean ± SD. RBC = red blood cell, WBC = white blood cell, MCV = Mean cell volume, MCH = Mean cell hemoglobin, MCHC = Mean corpuscular hemoglobin concentration. Distinct lowercase superscripts in the rows show significant differences (*p* < 0.05).

**Table 7:** Body composition and organosomatic indices of Nile tilapia (*O. niloticus*) fed diets supplemented with EPS for 90 days.

Parameter	T1 (0.0 g/kg)	T2 (0.1 g/kg)	T3 (0.2 g/kg)	T4 (1.0 g/kg)	T5 (2.0 g/kg)
Crude protein (%)	62.82 ± 8.18	64.70 ± 6.09	61.52 ± 6.14	65.06 ± 9.01	69.41 ± 8.22
Crude lipid (%)	4.14 ± 0.38	3.45 ± 0.23	3.87 ± 0.27	3.77 ± 0.03	3.68 ± 0.13
Moisture (%)	3.35 ± 0.07	3.23 ± 0.04	3.72 ± 0.05	3.35 ± 0.00	3.13 ± 0.01
Ash (%)	5.50 ± 0.25	5.59 ± 0.30	5.58 ± 0.11	4.64 ± 0.90	4.57 ± 0.30
Fillet (%)	33.48 ± 2.14	34.16 ± 0.46	35.48 ± 11.04	33.07 ± 2.24	35.55 ± 4.74
Carcass (%)	48.66 ± 2.70	46.12 ± 2.67	55.05 ± 2.13	50.56 ± 12.17	47.61 ± 4.91
Hepatosomatic index (HIS; %)	2.42 ± 0.58	2.62 ± 0.72	2.78 ± 0.91	2.62 ± 0.55	2.71 ± 0.53
Viscerosomatic index (VSI; %)	10.54 ± 1.77	10.91 ± 1.80	11.91 ± 3.57	10.47 ± 1.57	10.82 ± 1.32
Spleenosomatic index (SSI; %)	0.20 ± 0.05	0.18 ± 0.050	.25 ± 0.13	0.27 ± 0.03	0.21 ± 0.03

Data are given as mean ± SD.

**Table 8:** Blood biochemical profiles of Nile tilapia (*O. niloticus*) fed diets supplemented with EPS for 90 days.

Parameter	T1 (0.0 g/kg)	T2 (0.1 g/kg)	T3 (0.2 g/kg)	T4 (1.0 g/kg)	T5 (2.0 g/kg)
Total protein (g/dL)	2.66 ± 0.15	3.00 ± 0.26	2.70 ± 0.17	3.00 ± 0.36	2.90 ± 0.10
Albumin (g/dL)	0.83 ± 0.05	0.80 ± 0.10	0.80 ± 0.02	0.90 ± 0.10	0.86 ± 0.05
Globulin (g/dL)	1.83 ± 0.15	2.20 ± 0.17	1.90 ± 0.17	2.10 ± 0.26	2.03 ± 0.05
Aspartate aminotransferase; AST (U/L)	73.66 ± 14.70	56.33 ± 16.66	69.33 ± 13.12	36.33 ± 15.30	54.66 ± 19.32
Alanine aminotransferase; ALT (U/L)	39.33 ± 8.62	30.33 ± 16.01	48.33 ± 18.36	30.00 ± 10.15	55.66 ± 15.55
Blood Urea Nitrogen; BUN (mg/dL)	1.33 ± 0.57	1.50 ± 0.50	1.16 ± 0.28	1.33 ± 0.29	1.35 ± 0.57
Cholesterol (mg/dL)	123.66 ± 6.50	130.66 ± 10.21	117.33 ± 29.31	108.66 ± 21.34	134.00 ± 11.35

Data are given as mean ± SD.

**Table 9:** Immunological and antioxidant parameters of Nile tilapia (*O. niloticus*) fed diets supplemented with EPS for 90 days.

Parameter	T1 (0.0 g/kg)	T2 (0.1 g/kg)	T3 (0.2 g/kg)	T4 (1.0 g/kg)	T5 (2.0 g/kg)
Lysozyme activity (U/mL)	5.80 ± 2.77 ^b^	7.30 ± 2.10 ^b^	11.00 ± 2.54 ^a^	11.52 ± 2.21 ^a^	12.16 ± 1.68 ^a^
Myeloperoxidase; MPO (OD_450nm_)	2.00 ± 0.74 ^b^	1.76 ± 1.15 ^b^	2.59 ± 0.84 ^ab^	3.28 ± 0.17 ^a^	3.21 ± 0.19 ^a^
Superoxide dismutase; SOD (U/g liver)	10.20 ± 1.48 ^c^	14.20 ± 0.83 ^b^	11.60 ± 3.64 ^bc^	16.60 ± 2.40 ^b^	20.80 ± 3.49 ^a^
Catalase activity; CAT (U/g liver)	26.25 ± 9.27 ^c^	31.25 ± 9.88 ^bc^	39.06 ± 9.37 ^abc^	46.25 ± 11.35 ^a^	42.50 ± 5.22 ^ab^
Malondialdehyde; MDA (µmol/L)	77.82 ± 8.90 ^a^	77.35 ± 6.75 ^a^	82.88 ± 20.42 ^a^	75.94 ± 7.92 ^a^	92.39 ± 10.10 ^a^

Data are given as mean ± SD. Different superscripts in the rows mean significant differences (*p* < 0.05).

## Data Availability

The datasets generated during and/or analyzed during the current study are available from the corresponding author upon reasonable request.
